# Calcium‐binding proteins in focal cortical dysplasia

**DOI:** 10.1111/epi.13053

**Published:** 2015-06-17

**Authors:** Giorgi Kuchukhidze, Anna Wieselthaler‐Hölzl, Meinrad Drexel, Iris Unterberger, Gerhard Luef, Martin Ortler, Albert J. Becker, Eugen Trinka, Günther Sperk

**Affiliations:** ^1^ Department of NeurologyMedical University of InnsbruckInnsbruckAustria; ^2^ Department of NeurologyChristian Doppler KlinikParacelsus Medical University of SalzburgSalzburgAustria; ^3^ Department of PharmacologyMedical University of InnsbruckInnsbruckAustria; ^4^ Department of NeurosurgeryMedical University of InnsbruckInnsbruckAustria; ^5^ Institute of NeuropathologyUniversity of Bonn Medical CenterBonnGermany

**Keywords:** Focal cortical dysplasia, Temporal lobe epilepsy, Calcium‐binding proteins

## Abstract

**Objective:**

Alterations in γ‐aminobutyric acid (GABA)‐ergic cortical neurons have been reported in focal cortical dysplasia (FCD)Ia/IIIa, a malformation of cortical development associated with drug‐resistant epilepsy. We compared numbers of neurons containing calcium‐binding proteins parvalbumin (PV), calbindin (CB), and calretinin (CR) and densities of respective fibers in lateral temporal lobe surgical specimens of 17 patients with FCD with 19 patients who underwent anterior temporal lobe resection due to nonlesional temporal lobe epilepsy (non‐FCD) as well as with 7 postmortem controls.

**Methods:**

PV‐, CB‐, and CR‐immunoreactive (IR) neurons were quantitatively investigated with use of two‐dimensional cell counting and densitometry (reflecting mainly IR fibers) in cortical layers II, IV, and V.

**Results:**

Numbers of PV‐IR neurons, ratios of PV‐containing to Nissl‐stained neurons (correcting for eventual cell loss), and densities of PV‐IR were higher in layer II of the cortex of FCD compared to non‐FCD patients. Similarly, densities of CB‐IR and CR‐IR were also higher in layers II and V, respectively, of FCD than of non‐FCD patients. Comparison with postmortem controls revealed significant higher cell numbers and fiber labeling for all three calcium‐binding proteins in FCD cortex, whereas numbers of Nissl‐stained neurons did not vary between FCD, non‐FCD, and postmortem controls. In non‐FCD versus postmortem controls, ratios of calcium‐binding protein‐IR cells to Nissl‐stained neurons were unchanged in most instances except for increased CB/Nissl ratios and CB‐IR densities in all cortical layers.

**Significance:**

Increased numbers of PV neurons and fiber labeling in FCD compared to nondysplastic epileptic temporal neocortex and postmortem controls may be related to cortical malformation, whereas an increased number of CB‐IR neurons and fiber labeling both in FCD and non‐FCD specimens compared with postmortem controls may be associated with ongoing seizure activity. The observed changes may represent increased expression of calcium‐binding proteins and thus compensatory mechanisms for seizures and neuronal loss in drug‐resistant epilepsy.

Focal cortical dysplasia (FCD) is a malformation of cortical development, which broadly falls within three categories. Type I FCD is characterized by radial and/or tangential dyslamination of the neocortex.^1^ It is histologically distinct from type II FCD, which is defined by the presence of dysmorphic and giant neurons as well as balloon cells, and type III FCD, which is associated with hippocampal sclerosis (IIIa), tumors (IIIb), vascular malformations (IIIc), or brain injury/infection (IIId).^1^ Type I FCD presents histologically with abnormal cortical layers affecting either radial (FCD Ia) or tangential (FCD Ib) organization of a six‐layered cortex.^1^ Histologic hallmarks of FCD Ia are “microcolumns” defined as more than eight neurons aligned vertically. The interface between gray and white matter is usually irregular due to numerous ectopic neurons located in the white matter.^1^

Temporal lobe epilepsy (TLE) is the most common focal epilepsy syndrome in adults. It is frequently associated with FCD type I, which is increasingly diagnosed in vivo due to advanced imaging techniques and magnetic resonance imaging (MRI) postprocessing.^2^, ^3^, ^4^ However, FCD type I is notorious for being missed on routine MRI, comprising up to 40% of cases with reported normal MRI results.^5^ FCD type I frequently affects temporal lobe and its typical MRI characteristics are focal loss of volume (often described as “shrinkage”) and mild increase in MR‐signal in T_2_‐weighted [especially in fluid‐attenuated inversion recovery (FLAIR)] sequences.^5^

TLE is commonly refractory to pharmacotherapy and it is presumed that one of the mechanisms of drug resistance is associated with the disturbance in GABAergic interneuron circuits in the neocortex and hippocampus of patients with TLE.^6^ Immunostaining of calcium‐binding proteins such as parvalbumin (PV), calretinin (CR), and calbindin (CB) has been utilized for assessment of subtypes of inhibitory GABAergic neurons in TLE. PV, CR, and CB belong to the so‐called EF‐hand family of calcium‐binding proteins. They reside in well‐defined subpopulations of neurons in many vertebrate species and represent “buffer” proteins responsible for shaping the amplitude and duration of calcium signals. Immunocytochemistry has been useful for studying the development of human cortex as well as alterations of neuronal circuits in human brain diseases and experimental models.^7^, ^8^, ^9^ In experimental FCD as well as human dysplastic neocortex, a reduction of PV‐labeled interneurons has been shown.^8^, ^9^ Despite these well‐established alterations in immunostaining of calcium‐binding proteins in FCD neurons, it is unclear whether these changes are associated with specific layers in the neocortex. It is also unknown whether the alteration in the density of calcium‐binding protein‐immunoreactive (IR) neurons is due to the neurobiology of FCD or whether it is determined by epilepsy features. A protective role of calcium‐binding proteins against excitotoxic insults has also been discussed in a number of experimental models of epilepsy and other neurologic conditions.^10^, ^11^, ^12^, ^13^, ^14^ Therefore, in this study, we aimed to do the following: (1) investigate density of calcium‐binding protein‐IR neurons in different cortical layers of the temporal neocortex of patients who underwent epilepsy surgery due to drug‐resistant TLE; (2) compare the density of calcium‐binding protein‐IR neurons and their ratios to neuronal density between FCD and nonlesional temporal neocortex (we also compared these two groups with nonepilepsy postmortem controls); and (3) correlate morphologic and neurochemistry data with clinical parameters.

## Methods

### Patients

All patients underwent presurgical evaluation for their drug‐resistant TLE at the Department of Neurology, Medical University of Innsbruck. The localization and extent of the possible epileptogenic zone was determined by seizure semiology, ictal and interictal electroencephalography (EEG), brain MRI, and when necessary, fluorodeoxyglucose positron emission tomography (FDG‐PET), and ictal and interictal single‐photon emission computer tomography (SPECT). All patients underwent unilateral standard anterior temporal lobe resection including mesial temporal structures between January 2003 and September 2010. Surgical removal of the presumed epileptogenic zone was clinically indicated in order to achieve seizure control in every case. All procedures were conducted in accordance with the Declaration of Helsinki.

The inclusion criteria for the core group (FCD) were unilateral TLE determined by electroclinical features and histopathologic diagnosis of either FCD Ia or FCD IIIa based on current International League Against Epilepsy (ILAE) classification.^1^ Patients with hippocampal sclerosis were not excluded. The histologic features of FCD did not differ in patients with (FCD IIIa) or without (FCD Ia) hippocampal sclerosis; therefore, we integrated these two types of cortical malformation in one group, naming it “FCD.” The “epilepsy” control group (non‐FCD) comprised patients with TLE and normal histology of the resected lateral temporal neocortex.

### Clinical, EEG, and MRI data

Demographic and clinical variables were obtained at the time of admission for epilepsy surgery. Seizure types throughout the whole duration of epilepsy, seizure frequency during the first year of epilepsy, as well as occurrence of epileptic seizures and their types during 72 h before epilepsy surgery were analyzed. All patients underwent at least one high‐resolution MRI using a standardized protocol at our institution.^15^

Twelve (70%) of 17 patients with histologically proven FCD Ia were suspected to harbor FCD on preoperative MRI. In total, 13 (36%) of 36 patients had hippocampal sclerosis (5/17, 29% in FCD group and 8/19, 42% in non‐FCD group), which was confirmed by the subsequent histopathologic examination in all cases.

### Histopathologic analysis

Neuropathologic analysis of the resected brain tissue was done at the Department of Pathology, Medical University of Innsbruck. The Institute of Neuropathology, University of Bonn, Germany, was consulted for the second opinion (AJB). Two patients with the insufficient surgical specimens preventing reliable histopathologic assessment were excluded from the analysis. The neuropathologists were blinded for clinical findings (except for epilepsy) and MRI diagnosis.

Surgical specimens were fixated in 10% neutral buffered formalin and processed in paraffin; afterwards they were serially cut perpendicular to cortical surface. The 4‐ to 10‐μm–thick slices were processed for conventional light microscopic study by using hematoxylin & eosin. FCD Ia was diagnosed if the “microcolumns” comprising more than eight vertically aligned neurons were seen and if the border between gray and white matter was irregular with multiple ectopic neurons in the white matter. Dysmorphic, cytomegalic neurons and balloon cells were not observed in the specimens. FCD IIIa was diagnosed when in patients with hippocampal sclerosis the architectural abnormalities in the temporal lobe were similar to histologic features of FCD Ia. FCD types were classified based on the current histologic nomenclature of FCD.^1^

### Tissue fixation and sectioning for histochemistry

All specimens were rinsed in 50 mm phosphate‐buffered saline (PBS, pH 7.4) at 4°C and photographed for documentation. For fixation, tissue blocks were immersed in 4% paraformaldehyde in PBS for 4–5 days, followed by stepwise immersion in sucrose (concentrations ranging from 5–20%) over 2 days. They were then immersed in −70°C isopentane for 3 min and put in a freezer at −70°C. After allowing the isopentane to evaporate for 24 h, tissue samples were sealed in vials and kept at −70°C. Prior to immunocytochemistry, microtome sections (40 μm) were collected and kept for up to 2 weeks in TBS/0.1% sodium acid at 8°C.

### Nissl staining

Nissl staining with cresyl violet (CV) was performed on 40 μm–thick mounted sections adjacent to those used for immunohistochemistry, as described in detail before.^16^

### Immunohistochemistry

The density of calcium‐binding proteins—PV, CB, and CR—was analyzed in neocortical specimens of temporal lobe obtained as a result of epilepsy surgery in two groups of patients: (1) with FCD type Ia/IIIa (FCD) and (2) with histologically intact temporal lobe neocortex (non‐FCD). As nonepilepsy control tissue, we utilized samples from the analogous regions of the temporal lobe neocortex obtained shortly postmortem at autopsies of seven patients (median age 54 years, interquartile range [IQR] 46–60, six men; mean postmortem time 10 h, range 8–13) who died due to causes unrelated to nervous system (pneumonia, lung cancer, pharynx cancer, breast cancer, liver cirrhosis, melanoma, and leukemia).

#### Antibodies

For PV, a monoclonal mouse antibody (1:4,000; P3088; Sigma, Vienna, Austria); for CB a monoclonal mouse antibody (1:10,000; 300; Swant, Marly, Switzerland); and for CR, a polyclonal rabbit antiserum (1:5,000; 7,698; Swant) was used. For detection of neuronal nuclear protein (NeuN), an unspecific marker contained in adult neurons a monoclonal mouse antibody (1:5,000; MAB 377, clone A60; Chemi‐Con, Millipore, Vienna, Austria) was used. As secondary antibodies, a biotinylated horse anti‐mouse (PK4002; Vector, Szabo‐Scandic, Vienna, Austria) and a biotinylated goat anti‐rabbit antibody (PK4001; Vector, Szabo‐Scandic) were used for labeling the primary mouse (NeuN, PV and CB) or rabbit (CR) antibodies, respectively.

#### Indirect immunohistochemistry

Adjacent 40 μm–thick free‐floating sections were used for indirect immunohistochemistry. ABC Standard test kit Vectastain (Vector Labs, Szabo‐Scandic) was used as described previously.^17^ Omission of the primary antisera served as control and eliminated specific immunoreactivities.

### Determination of numbers of calcium‐binding protein‐containing cells

**Figure 1:**
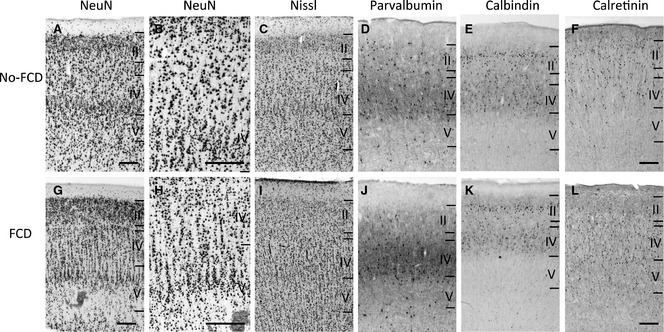
FCD and non‐FCD cortical sections. Representative cortical sections from non‐FCD (**A**–**F**) and FCD patients (**G**–**L**) that were Nissl stained (**C**,** I**) or labeled for NeuN (**A**,** B**,** G**,** H**), parvalbumin (PV;** D**,** J**), calbindin (CB;** E**,** K**), or calretinin (CR;** F**,** L**). For the orientation and definition of different cortical layers and cell counts we utilized the CB‐labeled sections. Note microcolumns of FCD cortex in G and H. The evaluated areas of the layers (II, IV, and V) are shown. Scale bars: 250 μm (in **A**,** F**,** G**,** L** for all panels except for **B** and **H**).

The numbers of PV‐, CB‐, and CR‐ stained neurons were counted at 100 times magnification. Layers II, IV, and V were evaluated separately using an ocular grid. In each section, IR neurons were counted in four different areas of each layer, each comprising 0.4 mm^2^ (40 caskets). For adjusting the layers, we oriented at the CB‐IR sections and defined the layers as shown in Figure 1. Using the ocular grid, sectors underlying 4× 10 caskets (0.4 × 1 mm) were counted for each layer in each of four areas in each section. Numbers of four areas were averaged. Numbers of cresyl violet–positive neurons were counted in the same way at 200 times magnification in sectors of 0.2 mm^2^ (8 × 10 caskets, corresponding to 0.4 × 0.5 mm), in four different areas of each section and averaged. From these numbers, median values were then calculated for all specimens of three groups (FCD, non‐FCD, and postmortem controls).

### Semiquantitative assessment of total calcium‐binding protein immunoreactivities in different cortical layers

We assessed calcium‐binding protein IR in different cortical layers for obtaining an estimate of possible changes in densities of calcium‐binding protein containing neuronal fibers. The resulting values represent primarily fiber labeling and, only to a minor degree, labeled neurons. We digitalized five cortical sections of FCD, non‐FCD, and postmortem specimens at 50 times magnification under a microscope equipped with a digital camera (ORCA‐ER; Hamamatsu Photonics Deutschland GmbH, Germany). Using the open source software IMAGEJ (ImageJ 1.47v; NIH, U.S.A., http://imagej.nih.gov/ij) a line‐scan (width: 500 pixels) over cortical layers was performed and obtained gray values were saved for further analysis. Values for background labeling were obtained from the white matter. Relative optical densities (RODs) were calculated from gray‐scale values using the formula [ROD = −LOG (gray value/256)] and background labeling was subtracted.

Optical densities of line scans obtained for the individual calcium binding protein‐IR and for the individual layers were analyzed using PRISM (GraphPad Software Inc., La Jolla, CA, U.S.A.). Data points for individual layers were summed up and statistical analysis was done by two‐way ANOVA.

### Statistical analysis

Categorical data were analyzed by means of 2 × 2 chi‐square test with Yates correction. Either Freeman‐Halton extension of Fisher's exact probability test or chi‐square test with Yates correction was used for tables larger than 2 × 2. In case of significant differences, paired‐wise comparisons were carried out by means of 2 × 2 Fisher's exact probability test or 2 × 2 chi‐square test with Yates correction. Noncategorical data (e.g., age at seizure onset, density of calcium‐binding protein immunostained neurons) were first analyzed by Kruskal‐Wallis test. Post hoc multiple comparisons following Kruskal‐Wallis test were performed by means of either Dunn's test or Mann‐Whitney *U* test. Significance was set at α = 0.05. There were no missing data in the entire analysis.

Results of a preliminary analysis (series of univariate ANOVA) indicated that age at seizure onset and age at the time of epilepsy surgery were not significant confounding factors with regard to the number of calcium‐binding IR neurons if FCD and non‐FCD groups were compared. The preliminary analysis also showed that absolute numbers of calcium binding IR neurons and their ratios to CV were not different if cases with and without hippocampal sclerosis were compared in both FCD and non‐FCD groups.

### Ethical issues

All patients provided written informed consent for the utilization of the resected brain tissue for research purposes. This study is a part of a large basic research project related to the human epileptic brain tissue, approved by the ethics committee of the Medical University of Innsbruck.

## Results

### Patients

Eighteen patients with FCD and 20 patients with non‐FCD met the aforementioned criteria from the database of >200 patients who underwent epilepsy surgery between 2003 and 2010 at the Department of Neurosurgery, Medical University of Innsbruck. One patient from each group was excluded from the analysis due to insufficient surgical specimens preventing reliable immunohistochemical assessment. Eventually, 17 patients with FCD and 19 patients with non‐FCD entered the final analysis. The FCD group included nine women and eight men with median age 25 years (IQR 21–37). In the non‐FCD group, there were 11 women and 8 men, median age 42 years (IQR 34–52).

Table 1 presents clinical data for each patient. Patients with FCD compared to those with non‐FCD had earlier seizure onset (median age 5 vs. 17 years, p = 0.012) and were younger at the time of surgery (median age 25 vs. 42 years, p = 0.002). The time to epilepsy surgery was not different in both groups: 19 (IQR 12–28) years in FCD and 20 (IQR 12–37) in non‐FCD group.

**Table 1:** Demographic and clinical features of study patients

Sex	Age at surgery	Age at seizure onset (year)	Epilepsy duration (year)	Seizure types	HS	Seizure frequency at onset	Seizure frequency at surgery	AEDs at surgery
FCD								
M	31	29	2	SPS CPS sGTCS	No	Weekly	Weekly	LEV, CLB
F	16	1	15	SPS CPS	No	Sporadic	Weekly	LEV
M	38	1	37	CPS	No	Monthly	Monthly	CBZ, LEV
M	31	1	30	CPS sGTCS	No	Daily	Monthly	LEV, LTG
F	49	5	44	SPS CPS sGTCS	No	Monthly	Monthly	OXC
M	24	5	19	SPS CPS sGTCS	Yes	Monthly	Monthly	PBT, LEV
M	37	9	28	SPS CPS sGTCS	Yes	Monthly	Monthly	CBZ, PGB, CLB
M	21	15	6	sGTCSs	No	Monthly	Monthly	LEV, ZNS
F	39	14	25	SPS CPS sGTCS	No	Weekly	Weekly	LEV, CBZ, PBT
M	24	15	9	SPS CPS sGTCS	No	Sporadic	Daily	LTG, PGB
F	14	12	2	CPS sGTCS	Yes	Monthly	Monthly	OXC, LEV, CLB
F	19	4	15	SPS CPS sGTCS	No	Sporadic	Sporadic	LEV, OXC
F	23	6	17	SPS CPS sGTCS	No	Sporadic	Monthly	LTG, CLB
M	26	1	25	SPS CPS sGTCS	Yes	Weekly	Daily	PBT, OXC, LEV, LCM
F	25	5	20	SPS CPS sGTCS	No	Monthly	Weekly	CBZ, LEV
F	60	1	59	CPS sGTCS	No	Sporadic	Monthly	CBZ, LCM
F	19	7	12	SPS CPS sGTCS	Yes	Monthly	Monthly	OXC, PGB, CLB
Non‐FCD								
F	34	14	20	SPS CPS	No	Monthly	Monthly	CBZ, CLB
M	35	26	9	SPS CPS sGTCS	No	Monthly	Monthly	PHT, LEV
F	42	5	37	SPS CPS sGTCS	No	Sporadic	Sporadic	PBT, CLB
F	55	2	53	SPS CPS sGTCS	Yes	Monthly	Monthly	CBZ, LEV
M	37	17	20	SPS CPS sGTCS	No	Monthly	Weekly	CBZ, TPM
M	53	39	14	SPS CPS sGTCS	Yes	Monthly	Weekly	CBZ, LTG
F	27	12	15	SPS CPS sGTCS	No	Daily	Daily	PBT, TPM
M	53	25	28	SPS CPS sGTCS	Yes	Monthly	Monthly	LEV, LTG, CLB
F	29	28	1	CPS sGTCS	Yes	Weekly	Weekly	PGB
M	41	1	40	SPS CPS sGTCS	Yes	Daily	Daily	CBZ
F	52	26	26	SPS CPS sGTCS	Yes	Sporadic	Monthly	CBZ, ZNS, CLB
F	46	38	8	CPS sGTCS	Yes	Daily	Monthly	VPA, CBZ, PBT
M	44	12	32	SPS CPS sGTCS	No	Monthly	Monthly	PBT, LTG
F	37	8	29	SPS CPS	No	Sporadic	Daily	PBT, PGB, ZNS
F	46	4	42	SPS sGTCS	No	Monthly	Monthly	CBZ, LEV, TPM
F	33	21	12	SPS CPS sGTCS	No	Weekly	Daily	CBZ, LEV
M	49	5	44	SPS CPS sGTCS	No	Weekly	Monthly	TPM, LTG
M	27	17	10	SPS CPS sGTCS	Yes	Monthly	Daily	PGB, LTG
F	54	40	14	SPS CPS	No	Weekly	Weekly	CBZ, LCM

HS, hippocampal sclerosis; AEDs, antiepileptic drugs; FCD, focal cortical dysplasia; SPS, simple partial seizure; CPS, complex partial seizure; sGTCS, secondary generalized tonic–clonic seizure; LEV, levetiracetam; CLB, clobazam; CBZ, carbamazepine; LTG, lamotrigine; OXC, oxcarbazepine; PBT, phenobarbital; PGB, pregabalin; ZNS, zonisamide; LCM, lacosamide; PHT, phenytoin; TPM, topiramate; VPA, valproic acid.

All patients in both groups had unilateral TLE and the laterality (left/right) was also equally distributed in both groups: 65% of patients had left‐sided and 35% had right‐sided TLE. The rate of the ipsilateral hippocampal sclerosis was higher in the non‐FCD group (42%) versus FCD group (29%); however, the difference was not statistically significant (p = 0.502).

Seizure frequencies during the first 2 years after seizure onset as well as before the surgery did not differ across the groups. None of the patients in FCD group had any seizure 72 h before the epilepsy surgery. In the non‐FCD group, however, 42% had seizures before the operation. These were simple partial (5/8), complex partial (2/8), and secondary generalized tonic–clonic (1/8) seizures.

The majority of patients in both groups were on polytherapy of antiepileptic drugs (AEDs): FCD 88% versus non‐FCD 89% (p = 1.0).

### Calcium‐binding protein immunoreactive cells

**Figure 2:**
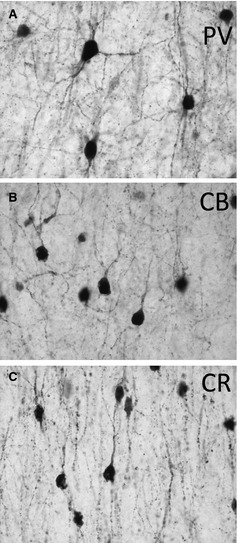
High magnification of parvalbumin (**A**), calbindin (**B**), and calretinin (**C**) immunoreactive neurons. Scale bar: 100 μm.

All three calcium‐binding proteins were well detected by immunohistochemistry in tissues originating from both groups of epilepsy patients (FCD and non‐FCD; Figs 1 and 2) and in postmortem controls (not shown). The antibodies for all three calcium‐binding proteins specifically labeled the respective interneurons and the associated fiber network (Figs 1 and 2). The regional distribution was well compared to that described by others.^18^, ^19^, ^20^ PV‐ and CB‐containing cells were about equally distributed in layers II through IV; the number of CR cells was somewhat higher in outer layers (Table 2, Fig. 1).

**Table 2:** Comparison of median values of calcium‐binding protein‐IR as well as cresyl violet–labeled neurons in the whole cortex and different cortical layers in FCD, non‐FCD, and postmortem controls

	FCD	Non‐FCD	p<	FCD	CTR	p<	Non‐FCD	CTR	p<
Total									
PV	116	89	***0.003***	116	61	***0.000***	89	61	ns
CR	186	164	ns	186	136	***0.000***	164	136	ns
CB	217	195	ns	217	90	***0.000***	195	90	***0.001***
CV	503	467	ns	503	436	ns	467	436	ns
Layer II									
PV	56	37	***0.002***	56	22	***0.000***	37	22	ns
CR	108	104	ns	108	92	***0.024***	104	92	ns
CB	103	95	ns	103	59	***0.000***	95	59	***0.003***
CV	188	194	ns	188	162	ns	194	162	ns
Layer IV									
PV	45	39	ns	45	30	***0.014***	39	30	ns
CR	43	36	ns	43	32	***0.009***	36	32	ns
CB	92	83	ns	92	20	***0.000***	83	20	***0.000***
CV	141	125	ns	141	125	ns	125	125	ns
Layer V									
PV	16	13	ns	16	9	***0.009***	13	9	ns
CR	31	21	ns	31	11	***0.000***	21	11	***0.005***
CB	18	15	ns	18	9	***0.000***	15	9	***0.010***
CV	174	147	ns	174	149	ns	147	149	ns

FCD, focal cortical dysplasia; CTR, postmortem controls; PV, parvalbumin; CR, calretinin; CB, calbindin; CV, cresyl violet; ns, not significant. Statistically significant differences are marked in bold and italic.

Table 2 summarizes median numbers of PV‐, CB‐, and CR‐IR neurons as well as CV‐positive cells in different patient groups. Calculations were performed separately in cortical layers (1) II; (2) IV; (3) V; as well as in the whole cortex (sum of all cortical layers). Densities of CV‐labeled neurons and of all calcium‐binding protein‐IR neurons did not differ across groups either in the whole cortex or separate cortical layers (Table 2). In the FCD group compared to the non‐FCD, a statistically significant increase in number of PV‐IR cells was observed in the whole cortex and layer II (p = 0.003 and p = 0.002, respectively; Table 2). There were no other significant differences in the density of assessed neurons (CV‐labeled and PV‐, CB‐, and CR‐IR) in different layers when FCD and non‐FCD groups were compared.

**Figure 3:**
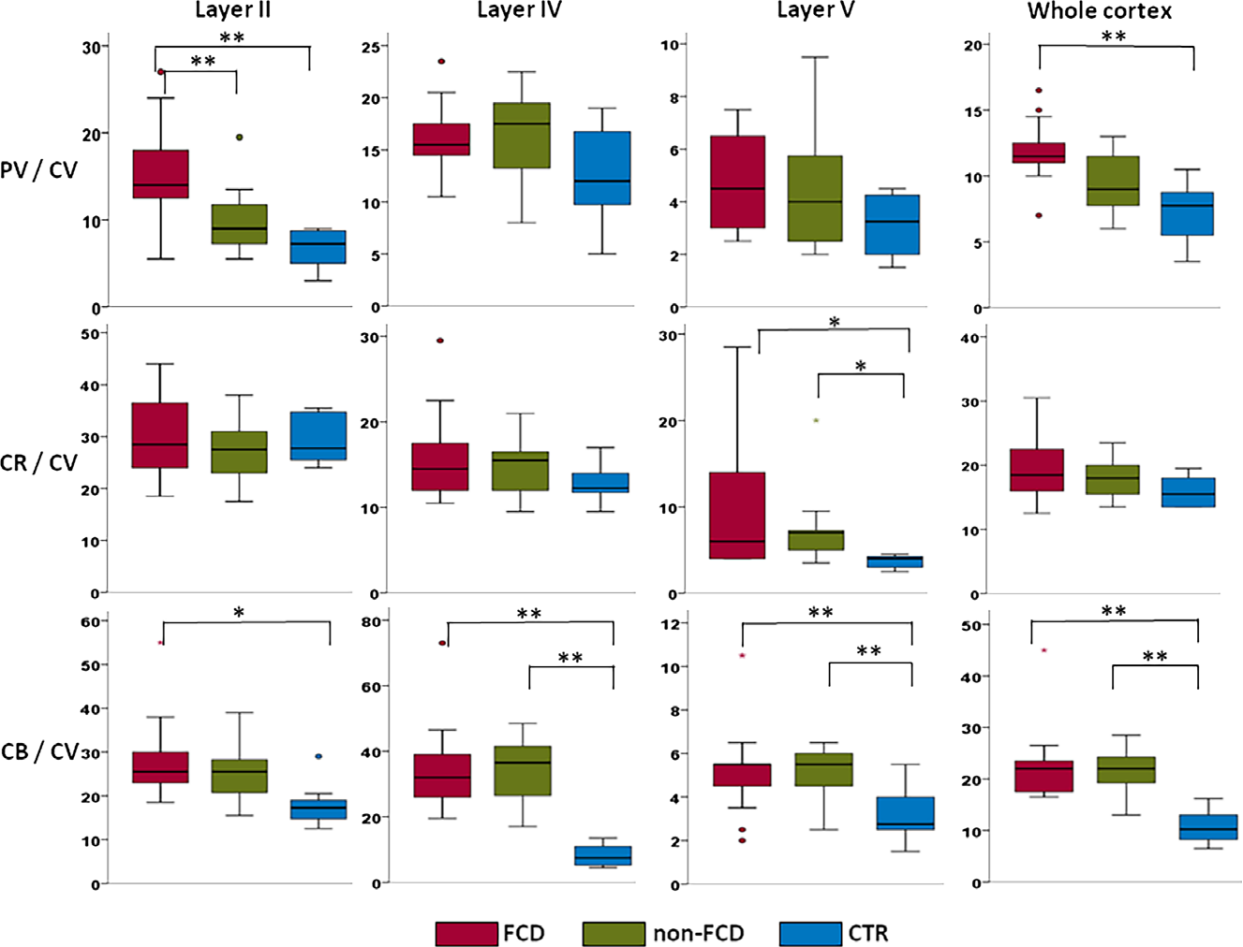
Ratios of calcium‐binding protein‐IR neurons to cresyl violet–labeled neurons in the whole cortex and different cortical layers in FCD, non‐FCD, and postmortem controls. Ratios are calculated from mean numbers of calcium‐binding protein‐immunoreactive neurons divided through the number of cresyl violet–labeled neurons. Statistical differences: *p between 0.01 and 0.05; **p < 0.01.

We then calculated ratios of numbers of calcium‐binding protein‐IR cells versus CV‐stained neurons (Fig. 3). The ratios of densities of PV‐IR to CV‐positive neurons were significantly higher in FCD specimens compared to non‐FCD and postmortem controls in layer II (p = 0.002 and p < 0.001, respectively). PV/CV ratio was also significantly higher in FCD compared to postmortem controls in the whole cortex (p = 0.001). In other layers across three groups the PV/CV ratios were not significantly different.

Ratios of densities of CB‐IR to CV‐positive neurons were significantly lower in all layers including the whole cortex in postmortem controls compared to FCD and non‐FCD (except for layer II where significant difference was observed only between FCD and controls). The CB/CV ratios did not differ across layers when FCD and non‐FCD groups were compared. Ratio of densities of CR‐IR to CV‐positive neurons was significantly lower in the layer V in postmortem controls compared to FCD and non‐FCD. The CR/CV ratios did not differ significantly across other layers when the three groups were compared.

### Total calcium‐binding protein‐IR representing primarily fiber labeling

**Figure 4:**
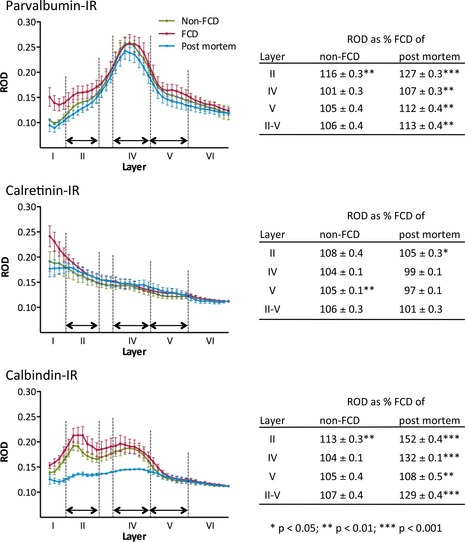
Line scans of calcium‐binding protein‐IR in cortical sections of FCD, non‐FCD, and postmortem specimens. Line scans were performed on digitalized sections (n = 5, for each group). Layers are indicated as defined for cell counts. Tables: Optical densities were integrated for each layer and presented as % FCD of non‐FCD and % FCD of postmortem controls, respectively, ± standard error of the mean (SEM). Statistical analysis was done by two‐way ANOVA. ***p < 0.001, **p < 0.01, *p < 0.05.

Total IR, representing mainly labeling of fibers, was highest for PV. Other than PV cell numbers, PV‐IR was most concentrated in layer IV, whereas CB‐IR peaked in layers II and IV and CR‐IR was slightly higher in outer than in inner layers (Fig. 4). In layer II, PV‐IR was significantly higher in FCD compared to non‐FCD (by 16 ± 0.3%; p < 0.01) and to postmortem controls (by 27 ± 0.3%; p < 0.001). CB‐IR was higher in layers II (by 13 ± 0.3%; p < 0.01) and IV (by 4 ± 0.1%; p < 0.05) and CR in layer V (by 5 ± 0.1%; p < 0.05) of FCD compared to non‐FCD. CR‐IR was considerably lower in all layers in FCD and non‐FCD.

## Discussion

The principal finding of this study is an increase in the number of PV‐IR neurons in the cortex of FCD compared with non‐FCD patients. This has been shown by increase in absolute numbers in the whole cortex and by increased ratios of PV‐containing neurons to Nissl‐stained neurons (normalized for eventual general cell loss) in the layer II and in the whole cortex. Comparison of calcium‐binding protein containing cells in FCD with postmortem controls revealed significantly higher numbers of neurons containing all of these three proteins (PV, CR, and CB). In contrast, the number of Nissl‐stained neurons was not different between FCD and postmortem controls in any cortical layer, indicating no general neuronal cell loss in the autopsy specimens. The changes in cell numbers, documented by cell counts for all three calcium‐binding proteins, were highly consistent with optical densities obtained by densitometric measurements in the different cortical layers. Optical densities primarily reflect changes in the density of IR fibers.

The higher numbers of immune‐labeled cells and IR‐fibers in FCD compared to postmortem specimens certainly could be due to postmortem degradation of the calcium‐binding proteins. On the other hand, the fact that in the autopsy samples neither the number of PV‐IR neurons, nor that of CR‐IR neurons (except of CR‐IR neurons in layer V) was significantly reduced compared with the non‐FCD specimen is in favor of postmortem stability of the calcium‐binding proteins. Of interest, however, the numbers of CB‐IR neurons were significantly higher in all cortical layers of FCD and non‐FCD specimens compared to postmortem controls. This difference was even more striking in densitometric measurements reflecting density of CB‐IR fibers mainly. These observations indicate that increase in numbers of CB‐positive neurons and fiber labeling could be triggered by seizure activity rather than by the malformation. It could also be due to a considerable weaker labeling of CB‐IR due to rapid postmortem degradation. In contrast, the higher number of PV‐IR neurons and fibers in FCD compared to non‐FCD specimens indicates that this difference may be due to the cortical malformation per se and not to the epileptic seizures.

In general, labeling for all three calcium‐binding proteins in our study was strong, both in neurons and fibers of epilepsy specimens and postmortem controls (Figs 1 and 2). Their distribution patterns in different cortical layers compare well to those previously reported.^21^ We observed roughly twice as many CR and CB neurons than PV neurons on entire sections. This is in agreement with data reported for the auditory cortex.^19^ In a study on the regional distribution of all three calcium binding proteins in 14 different cortical areas Bu et al.^22^ reported about equal densities of PV and CB neurons and higher numbers for CR neurons. This study is also in line with data reported by Beasley et al.^18^ for the prefrontal cortex. Slight differences between these studies (and also ours) could be due to the use of different antibodies.

However, our results showing increased numbers and fiber labeling of PV neurons are in variance with some previously published observations. These reported decreased numbers of PV‐ and CR‐IR neurons in the temporal neocortex of patients with FCD compared to postmortem nonepilepsy and nondysplastic epilepsy controls.^8^, ^9^ These discrepancies may be explained, at least in part, by the fact that different types of FCD from different locations, as well as specimens from children at considerably lower age than our patients were investigated in the previous studies.^7^, ^8^, ^9^

It has to be noted that our FCD patient cohort was significantly younger at seizure onset and at the time of the epilepsy surgery compared to the non‐FCD patients. However, these differences did not represent confounding factors with regard to the number of calcium‐binding protein‐IR neurons when FCD and non‐FCD groups were compared.

The presence of hippocampal sclerosis in both patient groups of our study did not influence the IR of calcium binding proteins in the neocortex. The expression of some proteins (e.g., GABA and glutamate transporters)^23^, ^24^ may be either similar in sclerotic versus nonsclerotic hippocampi^23^ or decreased in hippocampal sclerosis.^24^ In one of the few studies on altered neocortical properties in mesial TLE, it has been shown that the expression of α3‐GABA_A_ receptors is similar in patients with and without hippocampal sclerosis.^25^

We are presenting here evidence for changes in the number of PV‐containing neurons and labeling of respective fibers in cortical layer II of FCD patients. We can only speculate on possible underlying mechanisms. It may be possible that during embryonic development that the actual numbers of PV neurons become increased. Another possibility may be that the expression level of PV within GABA neurons may be increased, possibly due to increased activity of these neurons. This would lead to improved recovery of PV‐IR in immunohistochemistry. In line with this idea, we recently demonstrated in rats a loss in PV‐containing neurons in the entorhinal cortex and subiculum after kainic acid–induced status epilepticus.^26^ The surviving PV neurons, however, revealed markedly increased expression of PV‐mRNA (messenger RNA) due to the recurrent seizures. Thus, this finding indicates increased activity of surviving PV/GABA neurons aiming to compensate for the loss of functionally important neuronal population.^6^, ^13^

In addition to a locally altered activity in the dysplastic circuitry, ongoing epileptic seizures may trigger increased expression of calcium‐binding proteins. Such a mechanism seems, however, more likely for CB‐containing neurons than for PV neurons, since the number of CB neurons was increased in both FCD and non‐FCD specimens, whereas that of PV neurons was elevated only in the FCD samples. Calcium‐binding proteins are thought to be involved in buffering the intracellular calcium concentration and thus may counteract an intracellular “over‐load” with calcium. Thus, upregulation in calcium‐binding proteins protects neurons from overexcitation and neuronal damage.^10^, ^11^, ^12^, ^14^

In summary, we demonstrated novel changes in the expression of calcium‐binding proteins in FCD Ia/IIIa. Increased expression of PV‐IR in FCD compared to nondysplastic epileptic temporal neocortex and postmortem controls may be related to the cortical malformation, whereas the increased number of CB‐IR neurons in FCD and non‐FCD specimens may be related to ongoing seizure activity. Both changes may represent possible compensatory mechanisms for seizures and neuronal loss in drug‐resistant epilepsy.

## Disclosure of Conflict of Interest

GK received research support from FWF (Fonds zur Förderung der wissenschaftlichen Forschung), Austrian Science Fund (project number P 21636‐B18). AW‐H received research support from FWF (project numbers P 21636‐B18 and I 644‐B09). ET received research support from FWF (project numbers P 21636‐B18, KLI‐12, and P 24367), the European Union (E‐PILEPSY), and Red Bull Inc. ET received speaker honoraria from UCB Pharma, GlaxoSmithKline, Medtronics, Gerot Lannach, Bial, Newbridge, and Eisai; he served on scientific advisory boards for UCB (2005‐present), Eisai (2007‐present), J&J (2009), and Medtronics (2009–2011); ET received travel support by UCB Austria (2008) as well as travel expenses and honoraria for lectures or educational activities not funded by industry. ET has been a member of an editorial advisory boards of *Epilepsia* (2007–2011); *Epileptic Disorders* (2006–2012); *Zeitschrift für Epileptologie* (2006‐present); *Therapeutic Advances in Neurological Disorders* (2008–2012); *Zeitschrift für klinische Neurophysiologie* (2010‐present), and Mitteilungen der Österreichischen Sektion der ILAE (2003‐present). GL received speaker honoraria from UCB Pharma and GlaxoSmithKline and Johnson & Johnson, he served on scientific advisory boards for UCB (2008‐present), Eisai (2007‐present) and Johnson & Johnson (2006). GL received travel support from UCB Austria (2008‐present) and Eisai Austria (2011‐present). IU, MO, MD, AJB, and GS have no disclosures. We confirm that we have read the Journal's position on issues involved in ethical publication and affirm that this report is consistent with those guidelines.
